# Guidance on using real-world evidence from Western Europe in Central and Eastern European health policy decision making

**DOI:** 10.57264/cer-2022-0157

**Published:** 2023-03-02

**Authors:** Bertalan Németh, Maria Kamusheva, Zornitsa Mitkova, Zsuzsanna Ida Petykó, Antal Zemplényi, Maria Dimitrova, Konstantin Tachkov, László Balkányi, Marcin Czech, Dalia Dawoud, Wim Goettsch, Rok Hren, Saskia Knies, László Lorenzovici, Zorana Maravic, Oresta Piniazhko, Spela Zerovnik, Zoltán Kaló

**Affiliations:** 1 Syreon Research Institute, Budapest, HU 1142, Hungary; 2 Department of Organization & Economics of Pharmacy, Faculty of Pharmacy, Medical University of Sofia, Sofia, BG 1000, Bulgaria; 3 Center for Health Technology Assessment & Pharmacoeconomics Research, Faculty of Pharmacy, University of Pécs, Pécs, Hungary; 4 Medical Informatics R&D Center, Pannon University, Veszprém, HU 8200, Hungary; 5 Department of Pharmacoeconomics, Institute of Mother & Child, Warsaw, PL 01-211, Poland; 6 Science Policy & Research Programme, Science Evidence & Analytics Directorate, National Institute for Health & Care Excellence (NICE), London, United Kingdom; 7 Cairo University, Faculty of Pharmacy, Cairo, Egypt; 8 Division of Pharmacoepidemiology & Clinical Pharmacology, Utrecht University, Utrecht, The Netherlands; 9 National Health Care Institute, Diemen, NL 1120 AH, The Netherlands; 10 Faculty of Mathematics & Physics, University of Ljubljana, Ljubljana, Slovenia; 11 Syreon Research Romania, Tirgu Mures, RO 540004, Romania; 12 G. E. Palade University of Medicine, Pharmacy, Science & Technology, Tirgu Mures, RO 540142, Romania; 13 Digestive Cancers Europe, Brussels, BE 1040, Belgium; 14 HTA Department of State Expert Centre of the Ministry of Health of Ukraine, Kyiv, Ukraine; 15 Ministry of Health, Ljubljana, Slovenia; 16 Centre for Health Technology Assessment, Semmelweis University, Budapest, HU 1091 Hungary

**Keywords:** Central and Eastern Europe, guidance, health technology assessment, real-world evidence, transferability

## Abstract

**Aims:**

Real-world data and real-world evidence (RWE) are becoming more important for healthcare decision making and health technology assessment. We aimed to propose solutions to overcome barriers preventing Central and Eastern European (CEE) countries from using RWE generated in Western Europe.

**Materials & methods:**

To achieve this, following a scoping review and a webinar, the most important barriers were selected through a survey. A workshop was held with CEE experts to discuss proposed solutions.

**Results:**

Based on survey results, we selected the nine most important barriers. Multiple solutions were proposed, for example, the need for a European consensus, and building trust in using RWE.

**Conclusion:**

Through collaboration with regional stakeholders, we proposed a list of solutions to overcome barriers on transferring RWE from Western Europe to CEE countries.

## Plain language summary

Collecting real-world data and generating real-world evidence from it is becoming more important for making better decisions in healthcare. We investigated the main barriers which prevent using real-world evidence in Central and Eastern Europe, originally generated in Western Europe. After identifying the nine most important barrier, with the help of local experts we proposed solutions to overcome those barriers. Several possible solutions were proposed, many of them highlighting the need for a European consensus on these matters and building trust in new methods. Our results can hopefully serve as a guidance document to help overcome the barriers.

## Tweetable abstract

Research investigating ways to overcome barriers preventing Central and Eastern European countries from using Western European real-world evidence for healthcare decision making, using a multi-methods approach to create a list of solutions in collaboration with local stakeholders.

Health technology assessment (HTA) is a multidisciplinary process to support health policy and financial decision making by determining the value of a health technology at different points in its lifecycle using explicit evaluation methods [1]. Lower-income European countries, especially those from Central and Eastern Europe (CEE), have a greater need for such evidence-based decision-making processes, due to their worse health status and more limited budget compared with Western European (WE) countries, which increases the opportunity cost of making suboptimal decisions [2].

Real-world data (RWD) are observational data obtained from routine clinical practice, while real-world evidence (RWE) entails evidence obtained from the analysis of RWD. In this sense, RWE is evidence derived from RWD, and is being increasingly used for HTA purposes [3–6]. RWE can provide key inputs to HTA decision making when other sources of information, like various research studies may not be available [7].

With the growing availability of RWD, mostly as a result of wider use of electronic healthcare records, there is a great potential for widespread use of RWE. This is true even in the CEE region, for example in Poland [8–10]. However, a number of general challenges about using RWE for HTA exist [7,11,12] that may hinder the realization of the perceived benefits. Specifically in CEE countries, most decision makers prefer to rely on traditional sources of evidence such as randomized controlled trials (RCTs), or even on expert opinion rather than using RWE [13].

Generating RWE from RWD is a process that requires significant resources and adequate stewardship [14], which can be particularly challenging for CEE countries [15], which are typically late adopters of health technologies. Such lag provides the opportunity for making use of RWD collected in early adopter WE countries and then transferred to CEE countries. However, since it is implausible that the HTA offices in CEE will have access to the RWD collected in WE jurisdictions, they can only draw conclusions from the published RWE that was generated elsewhere, without being able to analyze the underlying data. Researchers have already started exploring the feasibility of transferring RWE to late technology adopter countries, for example, in the case of medical devices. These researchers concluded that the process of re-using RWE generated in WE is not fully implemented in the CEE region [16]. Therefore, a thorough investigation of the barriers preventing the utilization of RWE was a crucial first step.

The main goal of the European Commission funded HTx H2020 (Next Generation Health Technology Assessment) project is to create a framework for the Next Generation Health Technology Assessment (HTA) to support patient centered, societally oriented, real-time decision making on access to and reimbursement for health technologies throughout Europe. As part of HTx, our research aimed at first, identifying the key barriers preventing the application of RWE created in the WE countries for decision-making purposes in CEE countries [17,18]; and second, proposing solutions to overcome these barriers. A previous paper described the identification process of the barriers in detail [13].

We did not limit ourselves to studying only RWE used for relative effectiveness during our work. Accordingly, we conducted our research with other types of possible outcomes in mind as well, including *inter alia* cost data. We also did not limit the research to any subgroup of health technologies, as we wished to propose solutions that apply to RWE generated by a wide range of complex health technologies (e.g., pharmaceuticals and medical devices).

## Materials & methods

In order to conduct our research, we used a multi-method approach, where a four-step work plan was established. First, (step 1) a scoping review of the literature was conducted, followed by (step 2) a series of internal discussions and a 2020 webinar with 57 stakeholders from 12 different CEE countries. These two steps are described in detail in our previous paper [13], the current article presents the results of the survey and the workshop.

Seventeen barriers were identified through the first two steps, as described in our previous paper [13]. The opinion of the research group was that the 17 barriers to the transfer of RWE from WE to CEE jurisdictions resulting from the first two steps [13] varied greatly in importance, and prioritization was needed to focus our efforts on the most pressing issues in this field. Therefore, we conducted (step 3) a survey to identify the most important barriers, and finally, (step 4) we organized a workshop to discuss and finalize the proposed solutions to the barriers with CEE stakeholders. Throughout the research, the following grouping of the barriers was used, based on the first two steps [13]:

- Technical or organizational barriers;
- Regulatory barriers;
- Clinical and scientific barriers;
- Perceptional barriers.

**Figure 1.**
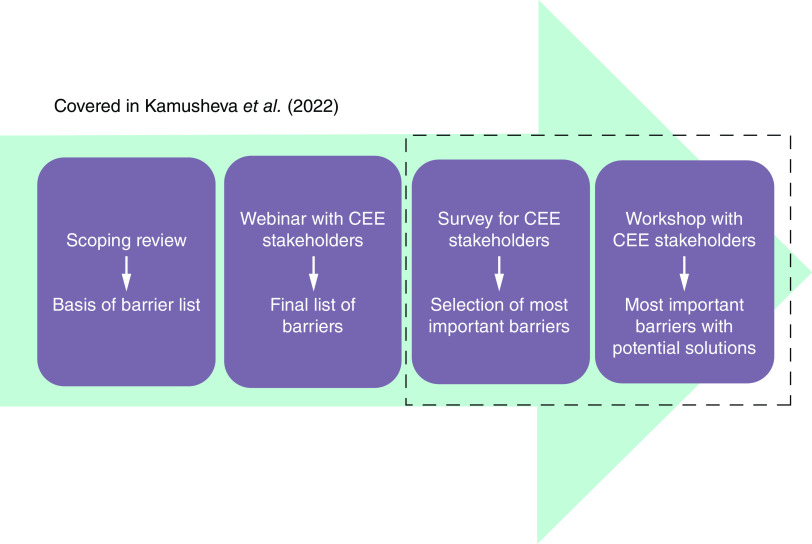
The workflow and publication process of our research. CEE: Central and Eastern Europe.

The workflow is demonstrated in Figure 1. The two key steps (step 3 and step 4) covered in this paper are highlighted with a dashed line.

The survey had a simple structure, as it listed all the 17 barriers to the transfer of RWE from WE to CEE jurisdictions that were the results of the first two steps (step 1 and step 2). The survey asked respondents to rate the barriers based on their importance (a Likert scale of 1–5) with the following explanation:

- Very low importance (1)
- Low importance (2)
- Medium importance (3)
- High importance (4)
- Very high importance (5)

The survey was first sent out to stakeholders who attended the webinar in step 2, who were mostly from the CEE region. Then, additional stakeholders with various affiliations also from the CEE region were asked to fill out the survey as well. Stakeholders were selected to represent as multiple CEE countries and as many affiliations as possible, and were assigned to one of the following categories:

-  Payer representative;
-  HTA organization representative;
-  Researcher or consultant or healthcare professional;
-  Health technology provider or manufacturer.

The survey results were assessed with two main principles in mind. First, the barriers that received high scores (above the median of all the mean scores) were considered important. Second, barriers that received lower than the median of all the mean scores but were considered highly important by at least one stakeholder group (a score of at least 4.00) were also added to the list of important barriers.

Based on the shortlist of the most important barriers and the learnings from the scoping review, researchers at Syreon Research Institute (Hungary) and the Medical University of Sofia (Bulgaria), who are also co-authors of this paper, proposed solutions to overcome the barriers. The solutions were to be discussed and finalized at the workshop in step 4.

The invitation-only workshop with CEE stakeholders was organized on 1 June 2022, as a satellite event of the 10th Adriatic and 7th Croatian Congress of Pharmacoeconomics and Outcomes Research in Pula, Croatia. Prior to the RWE workshop, the proposed solutions to the barriers (detailed recommendations) were sent to the invited participants to provide sufficient time for review and preparation The participants in the workshop were invited based on an iterative interaction with the professional networks of the HTx partners. The main selection criteria were familiarity with RWE-based HTA and policy decisions, also balancing participants by on their geographical location

Finally, participants were asked to take part in drafting this manuscript to ensure they were fully aligned with the proposed solutions to overcome the most important barriers.

## Results

**Figure 2.**
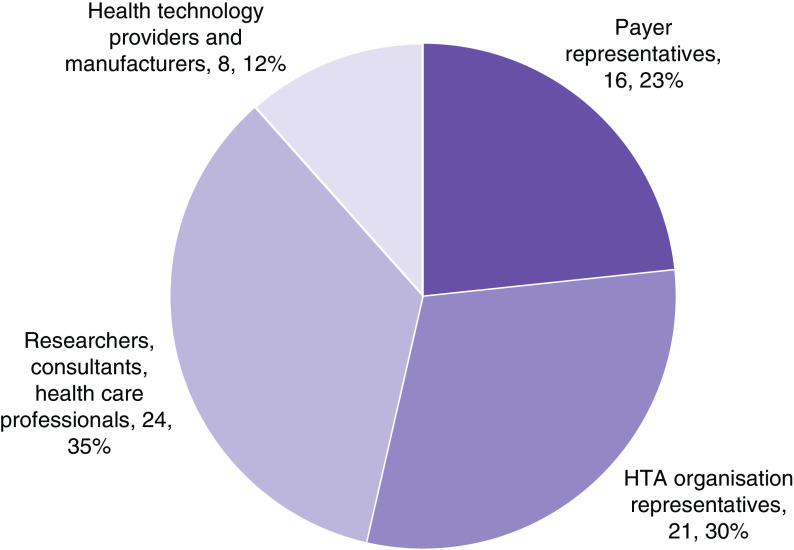
Participants by main affiliation. HTA: Health technology assessment.

**Figure 3.**
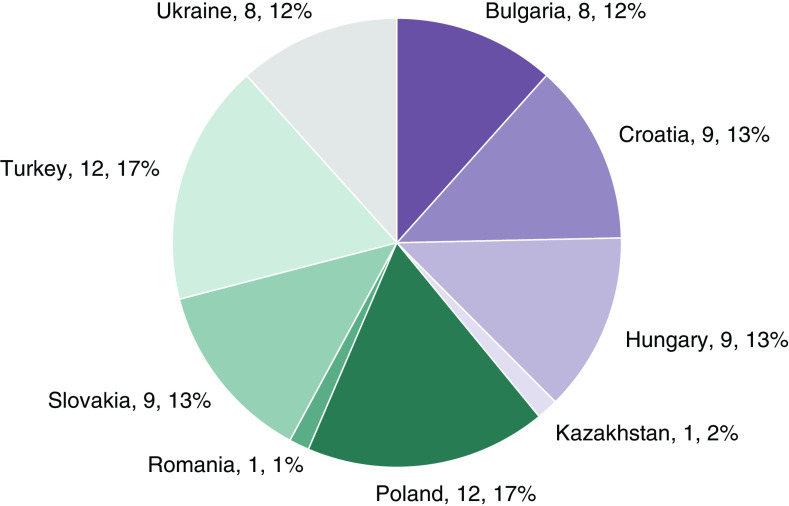
Participants by country represented.

A total of 69 CEE stakeholders filled out the survey in step 3. Of these, 16 responses came from payer representatives, 21 from HTA organization representatives, 24 from researchers and consultants and healthcare professionals and eight from health technology providers and manufacturers. The survey respondents represented the following countries: Bulgaria (8 respondents), Croatia (9), Hungary (9), Kazakhstan (1), Poland (12), Romania (1), Slovakia (9), Turkey (12) and Ukraine (8). Figures 2 & 3 show key participant characteristics.

Mean scores were simply calculated by dividing the sum of scores for rank with the number of respondents. Results from the survey are summarized in Table 1.

**Table 1.** Results of the survey of ranking 17 barriers to the transfer of RWE from WE to Central and Eastern European jurisdictions, which were identified by Kamusheva *et al.* (2022).

Rank	Barrier name	Barrier type	Mean score
**1**	**Lack of a favorable local/national governance framework related to using RWE**	**Regulatory**	**3.74**
**2**	**Lack of unified, widely accepted and implemented guidance documents for all EU countries on how to publish and share RWE**	**Regulatory**	**3.70**
**3**	**Lack of cooperation standards and data integration for common HTA across Europe**	**Regulatory**	**3.64**
**4**	**Lack of clear and accepted requirements on how and when to use RWE**	**Regulatory**	**3.59**
**5**	**Uncertainty of the relevance of RWE due to the lack of access to underlying RWD**	**Perceptional**	**3.58**
**6**	**Differences in medical practice for specific patient groups**	**Clinical and scientific**	**3.54**
**7**	**Lack of available financial resources for using RWE**	**Technical or organizational**	**3.46**
**8**	**Uncertainty in the quality of RWE**	**Perceptional**	**3.36**
9	Lack of expertise in the HTA agencies to critically evaluate RWE	Technical or organizational	3.33
10	Differences in HTA agencies perceptions and preferences for RWE	Perceptional	3.32
11	Variability of impact and importance of RWE in decision making in different CEE countries	Perceptional	3.30
**12**	**Differences in predefined criteria for evaluation of the effectiveness of medicines**	**Clinical and scientific**	**3.29**
13	Differences in epidemiological data across countries	Clinical and scientific	3.22
14	Variations in disease severity classification	Clinical and scientific	3.00
15	Unique demographic, racial, ethnic and genetic characteristics	Clinical and scientific	2.88
16	Requirements for using only local evidence in HTA	Regulatory	2.74
17	Frequently changing regulations on RWE	Regulatory	2.42

Barriers selected as highly important are highlighted in bold.

CEE: Central and Eastern Europe; EU: European Union; HTA: Health technology assessment; RWD: Real-world data; RWE: Real-world evidence.

Initially, the first eight barriers were selected based on the pre-set requirement of being higher than the median of all the mean scores. After reviewing the results in detail, it was revealed that the barrier differences in predefined criteria for evaluation of the effectiveness of medicines achieved a score of 4.00 in the stakeholder group of health technology providers and manufacturers. Therefore, it was added to the list of most important barriers. In total, nine ‘top barriers’ were selected based on the survey responses.

Of these, one barrier was technical or organizational (lack of available financial resources for using RWE), while two were perceptional barriers (uncertainty in the quality of RWE and Uncertainty of the relevance of RWE due to the lack of access to underlying RWD). Two barriers were from the clinical and scientific category (differences in medical practice limiting RWE transferability for specific patient groups and differences in predefined criteria for evaluation of the effectiveness of medicines), and the remaining four barriers selected were all regulatory barriers.

While drafting recommendations, it became clear that two of the nine barriers were quite similar, and will quite likely have the same solutions. Therefore, these two barriers (differences in medical practice limiting RWE transferability for specific patient groups and differences in predefined criteria for evaluation of the effectiveness of medicines) were considered as one from this point onwards.

The workshop had 14 participants, including six from the HTx project itself. The workshop participants represented the following countries: Hungary, The Netherlands, Poland, Romania, Serbia, Slovenia, Ukraine and the UK. Eight participants were researchers by their primary affiliation, while five represented HTA bodies, and one participant was a patient representative.

Three main components were observed throughout the detailed proposed solutions for multiple barriers: the need for international collaboration; the benefits of improving the knowledge of various stakeholders; and the importance of political support, to improve the uptake of RWE. The final detailed proposed solutions to the barriers, based on the consensus with the experts, are the following:

### #1 Solutions to overcome the lack of available financial resources for using RWE

In order to attract competent staff for RWD-RWE based HTA, we suggest increasing funding for HTA office employees, with increased duties used as a justification, to be used both for higher salaries per person and the employment of more staff. This can be done by, for example, increasing HTA submission fees, which should be covered by the manufacturers or the governmental budget allocated for HTA. In addition, to offer competitive salaries for employees of HTA bodies (and RWE experts should not be an exception) a sufficient budget should be established, allowing experts to attend scientific conferences, to follow relevant courses or receive training and other means to access research resources, as literature and other information services. Finally, if it is not feasible to conduct RWE analysis within the HTA office, then it should be considered to allocate budget to involve RWE experts from academic institutions in the HTA process, or perhaps to expand the capacity of expertise and help retain employees. It is advisable to provide/to allow combined affiliations (HTA office + academic institution) to attract skilled experts.

### #2 Solutions to overcome the lack of unified, widely accepted & implemented guidance documents for all EU countries on how to publish & share RWE

We suggest pressing for a European (or international) consensus on the mandatory dissemination and minimal structural and content standard requirements of RWE publication, either in reports of HTA agencies or in scientific journals. Special focus should be given RWE emerging from outcome-based risk-sharing agreements, while acknowledging challenges linked to their confidential nature. Additionally, we suggest the publication of a few best practice examples together with the guidance.

### #3 Solutions to overcome the lack of cooperation standards & data integration for common HTA across Europe

First, we suggest joint discussions resulting in publication of common standards on RWE generation (e.g., the DARWIN EU project [19]) and RWE dissemination. Second, the number of EU wide RWE utilization projects should be increased to facilitate transferability solutions. Finally, specific RWE cooperation should be established within the joint European HTA framework [20,21]. In case RWE are included in the submission files as part of the joint EU assessment, it should be considered by the assessors to take this into account, even if some countries are not interested in including RWE in their own assessments.

### #4 Solutions to overcome the lack of clear & accepted requirements of how & when to use RWE

We strongly suggest discussions with local stakeholders of health policy decision making, which can be followed by a joint declaration on the importance of appropriate RWE use in the CEE region. Additionally, joint international training should be organized for different stakeholders, presenting reference cases on efficiency improvements due to using RWE. Finally, a few best practices should be developed based on the most successful applications, from which general rules can be established on how and when to use RWE.

### #5 Solutions to overcome the lack of a favorable local/national governance framework related to using RWE

We suggest that experts and shared expertise should be involved in developing or improving the local guidelines connected to the topic of RWE. Also, a positive environment for gathering, harvesting RWD, generating and critically assessing RWE should be created, which will likely increase the trust in RWE generated elsewhere.

### #6 Solutions to overcome the differences in medical practice limiting RWE transferability for specific patient groups & the differences in predefined criteria for evaluation of the effectiveness of medicines

The generalizability of findings from various studies should be explored, with the help of clinical experts and epidemiologists to develop a data or indicator-based checklist for the transferability of RWE. Second, an international catalogue of predefined criteria should be established for the relative effectiveness assessment of health technologies to reduce heterogeneity across countries, paving the way for more efficient RWE use. Finally, if there is uncertainty regarding the transferability of RWE, a living approach should be suggested: first use what is already available, then revise this decision if necessary. These proposed solutions can be linked to activities in HTx or similar European projects aiming to reduce heterogeneity in healthcare decision making.

### #7 Solutions to overcome the uncertainty in the quality of RWE

We suggest developing or adapting checklists, best practice guidelines [22] and various other quality assessment methods with recommendations on the (re-)use of RWE. Specifically, since several checklists are already available but not yet used, selecting one and advocating its utilization would be preferable. In more general terms, the number of EU-wide RWE utilization projects should be increased to facilitate solutions for transferability.

### #8 Solutions to overcome the uncertainty of the relevance of RWE due to the lack of access to underlying RWD

We suggest creating an open, transparent database with RWE that can be used for HTA purposes by different stakeholders as part of the joint European HTA framework [20,21]. The aim should be to adhere to the FAIR principles, suggesting that scholarly output should be FAIR (findable, accessible, interoperable and reusable) [23], as much as possible. Achieving FAIR requirements implicates using standardized content, structured terminologies, like the International Classification of Diseases by the WHO for example. Additionally, if challenged, experts can point out that in CEE countries, there is generally no standard to re-assess individual data from RCTs either, yet the results of RCTs are accepted more easily.

## Discussion

As the previous paper described the barriers to the transfer of RWE from WE to CEE jurisdictions in great detail [13], we focused on the proposed solutions in the current article. Furthermore, we are proposing that there may be an ongoing paradigm shift that may result in the more frequent use and hopefully simultaneously in the improvement of RWE quality. For those reasons we wish to establish a set of futureproof recommendations.

Though in our study we did not limit our focus to RWE for only certain types of outcomes, we believe that the most considerable potential for improvement is in relative effectiveness data. Counter-intuitively, late technology adopter countries, like those in the CEE region, can benefit from being late because the evidence will already be generated elsewhere by the time the local appraisal processes start. Related to this, it was indicated during our workshop that several barriers identified in CEE countries are also present in WE countries, though in most cases, in a less severe form.

As mentioned earlier, certain details can be observed across our recommendations. Multiple recommendations call for international collaboration to overcome the different barriers, that can complement the efforts of initiatives mostly addressing the data availability issue like DARWIN-EU [19] or federated data networks like IMI EHDEN [24]. Researchers have already pointed out, that in the field of healthcare data, common problems require standardized solutions [25]. A recent study concluded that contemporary RWD policies showed great variation across HTA institutions [26].

The second crosscutting detail is the need to improve knowledge for various stakeholders. Not only related improving the use and reuse of RWE but also to trust on the scientific method of generating RWE and decisions made based on RWE. Part of this paradigm shift is a need for a change in the way stakeholders view the hierarchy of scientific evidence. Researchers used to place RWE toward the lower levels of the evidence hierarchy, below RCTs, systematic reviews or meta-analyses, for example [27]. While the traditional evidence pyramid mainly focuses on internal validity, RWE ensures external validity. As more focus is given to external validity because of a life-cycle approach compared with internal validity, the importance and relevance of RWE is growing. This can potentially create a situation of more knowledge increasing the quality of RWD and RWE, which can contribute to even improved knowledge of RWE and its uses. During the discussion at our workshop, the importance of using RWE, either on its own or as additional evidence to RCTs was emphasized.

Finally, the third common detail across several proposed solutions is the need for political will to improve the uptake of RWE at the country-level, financially or legally. The need to create positive feedback loops, for example, on the improvement of the quality and usability of the data are crucial.

The prerequisite for the successful transfer of RWE from WE to CEE jurisdictions is the transparency of RWE, which is corroborated by the joint RWE transparency initiative of the International Society for Pharmacoeconomics and Outcomes Research (ISPOR) and the International Society for Pharmacoepidemiology (ISPE) [28]. One of their key recommendations was the need for researchers to register their RWE studies on publicly available portals.

Another important consequence of this transfer is that beyond the strictly defined point of making the decision on reimbursement of a health technology, improved and continuous gathering of RWD and generating RWE, use and reuse have the potential to improve the feasibility of certain managed entry agreements [29] as well. For example, it is likely that with the proposed solutions implemented, applying ‘coverage with evidence’ development schemes – one of the most valuable tools for late technology adopter countries [30] – will become easier for CEE payers, reducing the uncertainty related to their decisions.

The perceived likelihood and feasibility of the real-life application of our proposed solutions vary to some degree. As mentioned above, in some cases efforts were already made, for example, through the DARWIN-EU [19] or the IMI EHDEN [24] initiatives, that will likely contribute to overcoming some barriers. On the other hand, proposed solutions that require local political will can make the process more difficult compared with the more science-based solutions.

The main limitation of our study is that though we kept a wide scope regarding the type of RWE, we focused mainly on transferability considerations of re-using RWE from WE countries in CEE countries. Several other issues (like cultural and political differences) can potentially arise when investigating RWE transfer across other regions, which consequently affect the proposed solutions to overcome the identified barriers. However, we believe that due to the general nature of our recommendations (e.g., the importance of international collaboration), these may be with appropriate tailoring applied in other regions as well.

Another limitation of our study is the relatively restricted number of CEE stakeholders involved in our research. However, in order to overcome this limitation, we were able to involve the major key opinion leaders from various CEE countries, for example, acting as heads of payer institutions or HTA offices. However, the limited number of stakeholders prevented us from drawing conclusions on the differences between CEE countries.

Future research should provide further specific recommendations for other cases of transferring RWE across other regions. Additionally, this guidance paper can serve as a first step, potentially followed by pilot projects in the CEE region, implementing our proposed solutions and evaluating the results. This may also improve the generalizability of our recommendations.

## Conclusion

Our research identified the most important barriers to the transfer of real-world evidence from WE to CEE jurisdictions with the overall goal to use real-world evidence in the decision-making process. Then, through strong collaboration with regional stakeholders, we proposed a comprehensive list of solutions to overcome each of the identified barriers. We expect that the lessons learned in our research will be used in real-life practice for the benefit of CEE countries.

## Future perspective

It is likely that throughout the next decade, the importance of RWE will continue to grow in all European countries. Hopefully, our recommendations will foster improvements in using RWE, thereby making it easier to transfer RWE from WE to CEE countries. Strengthening international collaboration, while also improving country level RWD and RWE generation, concurrently with increasing trust in RWE-based decision making, will hopefully lead to more appropriate healthcare decisions and more efficient use of resources. These improvements can also form the foundation of the use of innovative payment models, further benefiting the healthcare systems in Europe.

**Summary points**

- The importance of real-world evidence (RWE) for the use of health technology assessment is growing.
- It is unlikely that health technology assessment agencies in the Central and Eastern European region will have direct access to the real-world data generated in Western European countries.
- There are a number of actual barriers against reuse of RWE generated in Western European.
- Based on our previous work and a stakeholder survey, we selected the most important barriers.
- We drafted proposed solutions to overcome the eight most important barriers.
- The proposed solutions were discussed in great detail and a comprehensive list was finalized in collaboration with Central and Eastern European stakeholders at a workshop.
- Proposed solutions included the calls for international collaboration, improvement of knowledge on RWE and political support for more widespread use.
- Our results can hopefully serve as guidance document to overcome the barriers.
